# Consistent Implementation of Decisions in the Brain

**DOI:** 10.1371/journal.pone.0043443

**Published:** 2012-09-12

**Authors:** James A. R. Marshall, Rafal Bogacz, Iain D. Gilchrist

**Affiliations:** 1 Department of Computer Science/Kroto Research Institute, University of Sheffield, Sheffield, United Kingdom; 2 Department of Computer Science, University of Bristol, Bristol, United Kingdom; 3 School of Experimental Psychology, University of Bristol, Bristol, United Kingdom; McMaster University, Canada

## Abstract

Despite the complexity and variability of decision processes, motor responses are generally stereotypical and independent of decision difficulty. How is this consistency achieved? Through an engineering analogy we consider how and why a system should be designed to realise not only flexible decision-making, but also consistent decision implementation. We specifically consider neurobiologically-plausible accumulator models of decision-making, in which decisions are made when a decision threshold is reached. To trade-off between the speed and accuracy of the decision in these models, one can either adjust the thresholds themselves or, equivalently, fix the thresholds and adjust baseline activation. Here we review how this equivalence can be implemented in such models. We then argue that manipulating baseline activation is preferable as it realises consistent decision implementation by ensuring consistency of motor inputs, summarise empirical evidence in support of this hypothesis, and suggest that it could be a general principle of decision making and implementation. Our goal is therefore to review how neurobiologically-plausible models of decision-making can manipulate speed-accuracy trade-offs using different mechanisms, to consider which of these mechanisms has more desirable decision-implementation properties, and then review the relevant neuroscientific data on which mechanism brains actually use.

## Introduction

### The Neural Implementation of Decision-Making

Consider a simple binary decision-making task, such as the choice of making a saccadic eye movement to one of two possible flickering objects, one of which is the slightly brighter target (e.g. [1]). In this kind of task the participant is able to favour decision-accuracy at the expense of decision-speed, or vice-versa, according to the instructions or the reward structure induced by the experimental design. Delaying a response would allow the participant to accrue more information about the probability of the target being at a particular location, and hence improve decision accuracy, but this would inevitably have a cost in terms of speed of the response. Remarkably, however, decision implementation can be highly stereotypical, and independent of decision difficulty (e.g. [2]).

**Figure 1:**
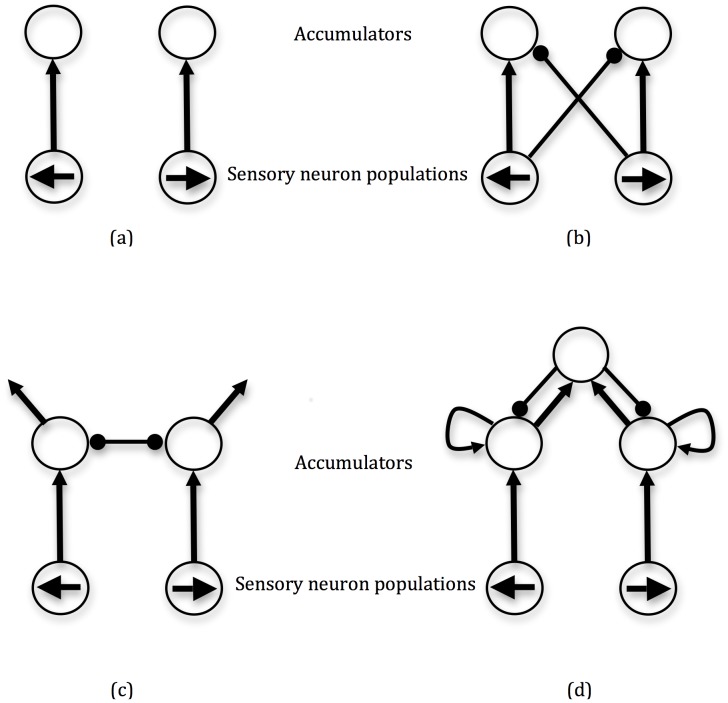
Accumulator models of decision-making. Sensory neuron populations for each decision alternative feed into corresponding accumulators, which must reach a threshold for an appropriate action to be initiated. Lines with arrows denote excitatory inputs, while circles denote inhibitory inputs. Arrowed lines with no target denote activation leakage from populations. (a) race model [14]. (b) feed-forward inhibition model [22]. (c) mutual inhibition model [5]. (d) pooled inhibition model [17].

This scenario is one that is simplified for experimental tractability, yet is sufficiently rich to illustrate general principles of decision-making. Accumulator models have been proposed that implement decision-making using competing neural populations that integrate evidence, with one accumulator for each alternative (e.g. [3]–[7]; figure 1). When an accumulator reaches a threshold, the decision is made for the corresponding alternative. For two alternatives, many such models can be parameterised to approximate the drift-diffusion model of decision making [8], which in turn realises the statistically optimal decision-making strategy, the Sequential Probability Ratio Test (SPRT), that minimises expected decision-time while obtaining a given error rate. Such models of decision-making account for neural activation patterns (see references earlier in the paragraph), give good fits to experimental data on decision accuracy and reaction-time distributions, and can be used to model a range of more complex tasks (e.g. [9]).

In accumulator models, the compromise between the speed and the accuracy of decision-making is typically achieved by adjusting the threshold that precipitates these decisions. It has been remarked, however, that for several classes of models, changing baseline activation of integrator populations has an equivalent effect (*e.g.* [10]). In the next section we illustrate that for a certain class of models manipulation of the threshold while holding fixed the baseline activation of the accumulators, and manipulation of the baseline activation while holding fixed the threshold, are formally equivalent. Researchers interested in the neural implementation of speed-accuracy trade-offs have therefore recently started to investigate which of these two alternatives is realised in the brain. Several recent studies, reviewed in [10], have presented emerging evidence for the baseline manipulation hypothesis, while little evidence has been presented for the threshold manipulation hypothesis to date.

While researchers have investigated whether the brain implements adjustable baseline activation, or adjustable thresholds, as yet there has been no principled explanation of why, at a computational level, one alternative is better than the other. We address this question by considering not only decision-making, but also the implementation of decisions reached. We then ask what characteristics would be desirable in a combined decision-making and implementation system, and how to design such a system.

## Results

### Equivalence Between Changing Threshold and Baseline Activation

In this section we explain how, for a certain class of models, lowering decision threshold and increasing the initial activation of accumulators produce the same changes to probability of error and reaction time distributions [11]. Thus, under such equivalence speed-accuracy trade-offs can be mediated in exactly the same way by changes in threshold or baseline activation, meaning that existing results showing that accumulator models with variable threshold between speed and accuracy instructions can fit behavioural data (e.g. [12]) imply that the models with variable baseline activation would describe these data equally well. Equivalence of these two mechanisms is also interesting because these models can be parameterised to implement statistically optimal decision-making, by approximating the drift-diffusion model described above [13]. This then allows us to consider the effect on *decision implementation* of choosing either variable baseline activation or variable thresholds, remaining confident that the dynamics of *decision making* are unaffected by the choice.

The equivalence just described is easiest to explain for the race model [14] which consists of two accumulators corresponding to two alternative choices (figure 1a). In each time step the activity of each accumulator increases proportionally to the input from a corresponding sensory neural population. The choice is made whenever the activity of any accumulator exceeds a threshold. In this model the time to reach the threshold depends on the difference between the initial activation of the accumulators at the stimulus onset and the threshold. Therefore reducing the threshold and increasing the initial activation have the same effects on model's behaviour. Similar logic applies to closely related models with independent accumulators like LATER [7] and the linear ballistic accumulator model [6].

For other models, the following two conditions are necessary for the equivalence of changing threshold and initial activation. First, the model needs to include separate accumulators corresponding to individual choice alternatives. Thus, the equivalence does not hold for the drift-diffusion model [8]. This model has a single accumulator integrating the difference between sensory evidence supporting the two alternatives. The choice is made when the activity of this single accumulator exceeds one threshold or falls below another threshold (situated below the initial state of the accumulator). In the diffusion model, changing the distance between thresholds affects the speed-accuracy trade-off, while changing the initial activation of the accumulator changes the bias towards choosing one or other of the alternatives. It is also difficult to conceive of a direct neurobiologically-plausible implementation of the drift-diffusion model, since it appears to require the possibility for negative activation levels of the accumulator. However, note that the diffusion model is an abstract model which can be implemented by embedding it in a more complicated, neurobiologically-plausible model [13]. For example in the feed-forward inhibition model (figure 1b; [15]), two accumulators act to integrate the difference between the input for the corresponding alternatives. Thus the activity of the first accumulator in the feed-forward inhibition model is proportional to the activity of the accumulator in the diffusion model, while the activity of the second accumulator is proportional to the negative of the activity of the accumulator in the diffusion model. In the feed-forward inhibition model, the choice is made when the activity of any of the accumulators exceeds a threshold, thus the model generates the same behaviour as the diffusion model. Nevertheless in the feed-forward inhibition model lowering the threshold and increasing initial activation produce equivalent changes to models' behaviour.

The second condition necessary for changes in the threshold and initial activation to be equivalent is the lack of non-linear terms in the equations describing the model. Thus the equivalence does not hold for the non-linear versions of the models shown in figures 1c and 1d [16]–[17], in which changing the range of activity in which the model operates very significantly changes the dynamics of the model. This is a limitation of our approach, since real neurons and neural populations are non-linear; however by considering linear models as abstract models of the underlying biology considerable progress has already been made in understanding the neural bases of decision making. For example linear accumulator models based on the feed-forward inhibition model (figure 1b) were successfully used to describe neural activity in lateral intraparietal area during decision making [15], [18]–[23], Furthermore, linear accumulator models based on the mutual inhibition model (figure 1c) were used to understand and map the functions of different neural populations in the frontal eye field during choice [24]. We return to this issue in the discussion.

**Figure 2:**
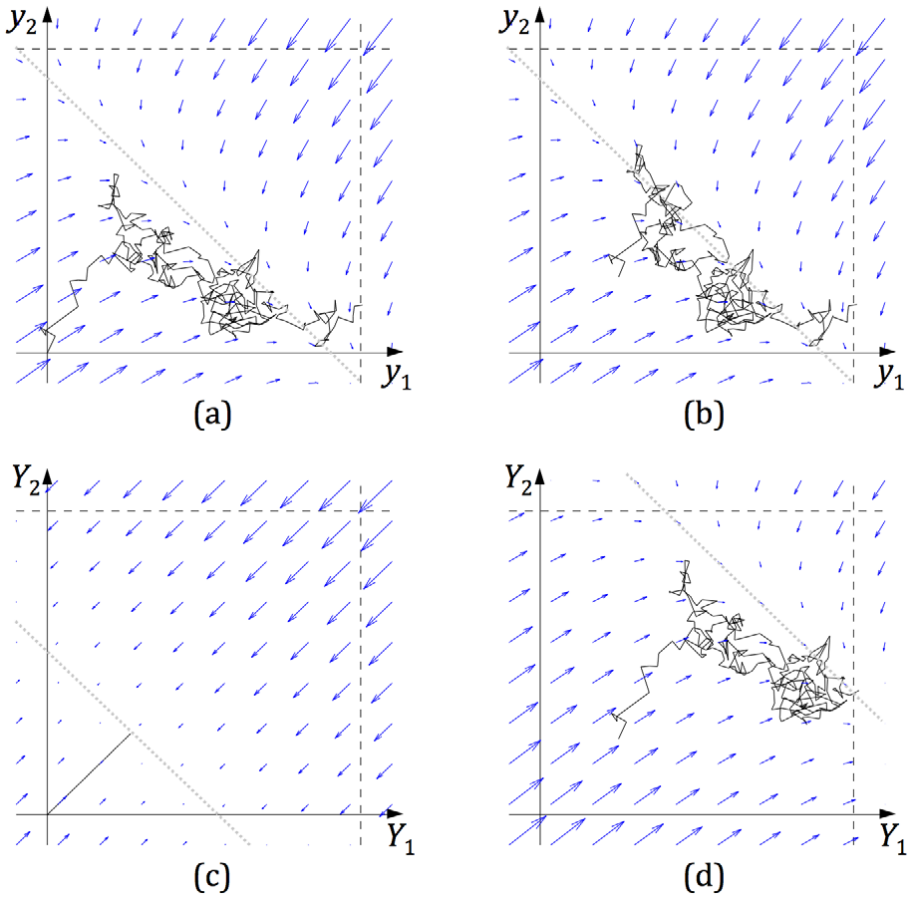
Dynamics of the mutual inhibition model (**equations A1** and **A2** )**.** In each panel the curve shows the evolution of the state of the model during a simulation, i.e. different points on the curve correspond to different time instances, and their co-ordinates correspond to levels of the activity of the accumulators at corresponding time. The simulations were performed using the Euler method with an integration constant of 0.001s. In all simulations *k* = *w* = 10, *I*_1_ = 4,41, *I*_2_ = 3, *c* = 0.33 (values of *I*_1_, *I*_2_, *c* were estimated from behaviour of a sample participant performing motion discrimination task as described in [13]) and the decision threshold was 0.4. The dashed lines indicate the positions in the state space in which one of the accumulators reaches a decision threshold. The arrows indicate the average direction in which the state moves from the point indicated by the arrow's tail, and its length corresponds to the speed of movement (i.e., rate of change) in the absence of noise. The dotted diagonal lines show the positions of the lines to which the state of the system is attracted. (a) Simulation of the model with *y*_1_(0) = *y*_2_(0) = 0. (b) Simulation of the model with *y*_1_(0) = *y*_2_(0) = 0.1. (c) Simulation of the model before stimulus onset (i.e. when *I*_1_ = *I*_2_ = *c* = 0). The simulation starts at *Y*_1_(0) = *Y*_2_(0) = 0, and the accumulators receive constant input *I*_0_ = 2 for 1s. (d) Simulation of the model with *Y*_1_(0) and *Y*_2_(0) set to the last state in panel c, in which the accumulators receive additional constant input of *I*_0_ = 2.

We now demonstrate that, in the linear version of the mutual inhibition model, lowering thresholds, and increasing baseline activity of accumulators by means of common input to both accumulators, give the same changes to the speed and accuracy of choices. The mutual inhibition model (figure 1c) includes two accumulators that integrate corresponding inputs until the activity of any of the accumulators reaches a threshold. Additionally, the accumulators inhibit each other and include leak (so their activity decays in the absence of inputs). Figure 2a shows a sample evolution of the activities of accumulators during a simulation. Initially both accumulators quickly increase their activities due to input (their trajectory moves from the origin up-right), but when they are sufficiently active they inhibit each other, so that when one increases its activity, the other decreases. Thus the state of the model slowly evolves along an attracting diagonal line (dotted line in figure 2a) until one of the thresholds is reached.

Since the mutual inhibition model includes leak, unlike race and feed-forward inhibition models, the baseline activity of the accumulators cannot be increased in a stable manner by simply setting the activities of accumulators to higher values, because the accumulators would quickly decay their values to 0 through leakage (in absence of any other input). Figure 2b illustrates that such a change in initial activities of accumulators also has almost no effect on the decision time, because this change only affects the movement towards the attracting line, but does not affect the position of the attracting line and hence evolution along it.

To increase the baseline activity of accumulators in a way that it is maintained, an additional input to both integrators needs to be provided before and during the decision process; this input could be achieved by increasing the base-level firing rate of neurons in the integrator populations. Figure 2c shows how the state of the system evolves when such an input is provided before stimulus onset (i.e. when no sensory input is provided). Although both accumulators continuously receive input, they do not increase all the time, because of leak and inhibition, and converge to the state when the input balances leak and inhibition. Figure 2d shows the simulation of decision-making in which the additional input is also provided throughout the choice process. This additional input shifts the position of the attracting line such that a smaller portion of the attracting line is between the thresholds, resulting in a faster decision.

All simulations in figure 2 were run with the same initial seed of the random number generator; note that the trajectories in panels 2a and 2d have the same shape but are shifted (as we show in Methods and Results). Therefore, shifting the trajectories towards thresholds by means of the common input to the accumulators has the same effect on the model's behaviour as lowering the thresholds in the mutual inhibition model.

The same shape of trajectories in figures 2a and 2d also implies that the additional input does not change the mean ‘rate of drift’ along the attracting line (see Methods and Results). As a result, decision-speed and accuracy are affected solely by the movement of the attracting line relative to starting activation levels of the accumulators, achieved by manipulating the common baseline input to both accumulators.

In Methods and Results we also show that the increasing baseline activity in the linear version of the pooled inhibition model (figure 1d) by means of increased input to accumulators has the same effect on the model's behaviour as lowering thresholds, for analogous reasons to those for the mutual inhibition model.

### Implementing Decisions

The majority of simple decisions lead to a motor response – in the example given in the introduction this is the generation of a saccade to one location or another. One desirable property of the combined decision process and response generation system is that the nature of the response generated should not depend on how the decision was reached. For example a decision that was made following only weak evidence in favour of that alternative should not result in a movement that itself is slow or inaccurate. Once the system has decided on a particular response, the response itself should be as accurate and as well executed as possible. This could be formulated as a minimisation problem, where the optimal implementation mechanism is that which minimises variability in decision implementation, across decision scenarios.

**Figure 3:**
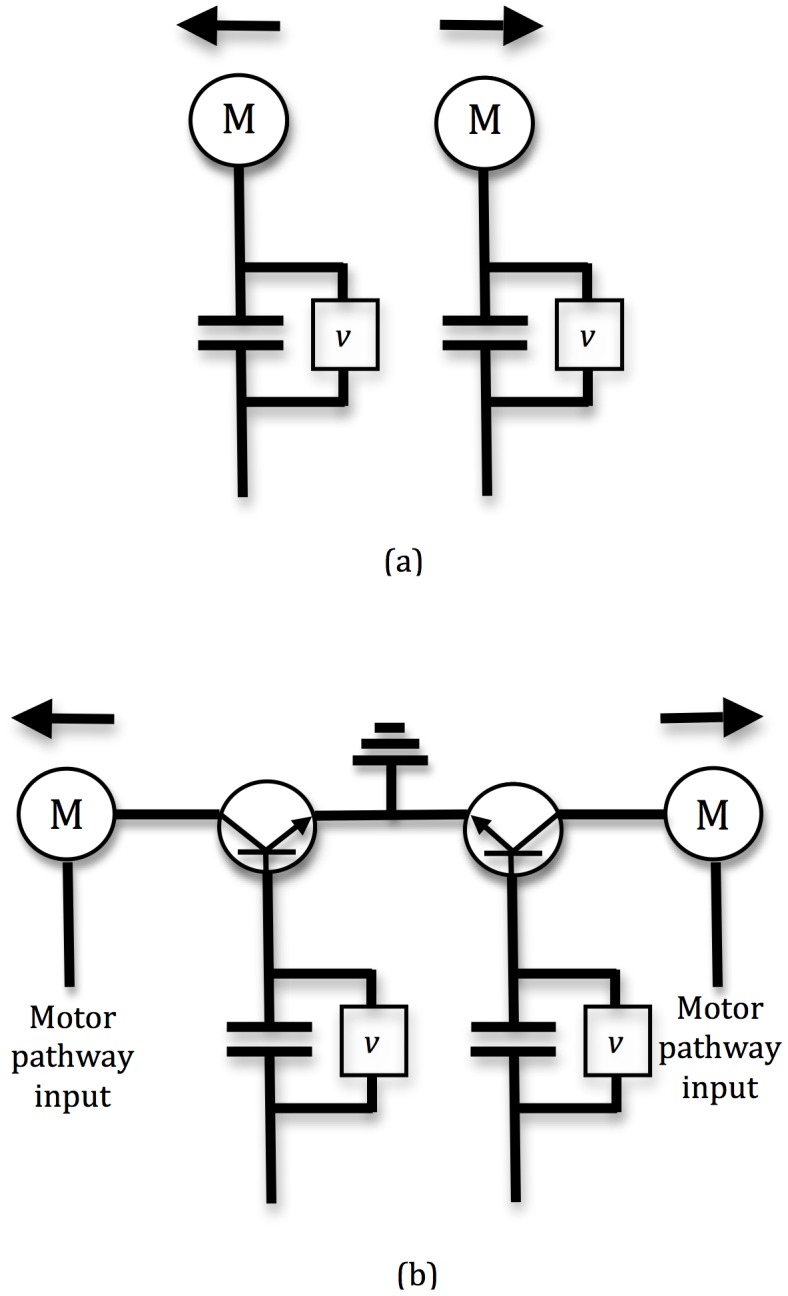
(a) An electrical circuit implementation of the race model (figure 1a). Noisy inputs for each decision alternative, in the form of fluctuating currents, are accumulated by capacitors. These capacitors continue to accumulate charge, until they reach a specified threshold (assessed by the circuit *v*). On reaching threshold, the capacitor discharges across a motor, which is taken to be the implementation of the decision reached. Variable capacitor thresholds result in variable inputs to the motor, according to decision type (low threshold, fast but inaccurate decisions result in weak motor movements, while high threshold, slow but accurate decisions result in strong motor movements). In contrast, holding capacitor thresholds constant but varying baseline capacitor charge realises consistent inputs to motors, and hence consistent decision implementation. (b) The circuit of (a), modified such that motor commands are implemented by disinhibiting a motor pathway. This is achieved by using the output from the capacitor corresponding to accumulated evidence for one alternative as the input to a transistor, which acts as a switch on the motor pathway. Since the current crossing a transistor varies as a function of its input, consistent outputs from the capacitor are also desirable in order to implement consistent motor actions.

We consider the interrelation between the decision and response processes with a simple analogy to the design of an electrical circuit for making decisions and then implementing corresponding motor actions. Figure 3 shows our circuit, designed to mimic the race model of decision-making shown in figure 1a. As with the race model we have two ‘accumulators’, which are actually capacitors in our circuit. Current flows into these at rates that vary randomly over time, such that nevertheless one of the currents, corresponding to the best decision alternative, is higher than the other on average. The capacitors thus accumulate charge over time. When one of the two decision-making capacitors reaches some threshold, the circuit should implement a decision. Thus far we have done no more than reproduce the race model as an electrical circuit diagram; we have chosen the race model for simplicity, but we could easily extend this basic model to implement something like the leaky competing accumulator model (figure 1c), with cross-inhibition and leakage from the capacitors. We now extend the original model by considering decision-implementation, represented in our model as connecting the output of each capacitor to a motor for the corresponding alternative. Each capacitor is accompanied by a simple control circuit (*v* in figure 3) that detects when its charge has reached the requisite threshold, and when it has done so fully discharges the capacitor across the motor.

Consider what happens to the circuit described above when making decisions under the adjustable decision-threshold model. For high decision-thresholds corresponding to slower, more accurate decisions, the capacitor reaching threshold first will contain a high charge, which will subsequently be discharged across the motor resulting in a large movement. For low decision-thresholds however, corresponding to quicker, less accurate decisions, the winning capacitor will contain a low charge, giving a small motion to the motor. Thus slow, accurate decisions lead to ‘powerful’ motor commands, while quick, inaccurate decisions lead to ‘weak’ motor commands. This dependence of motor behaviour on decision behaviour seems undesirable, and it is easily seen to be avoided under the adjustable baseline activation model. In this model, the baseline charge in the capacitors is changed while the activation threshold is kept fixed. Now slow, accurate decisions correspond to low baseline charge (or the common input to both capacitors; see Methods and Results), while quick, inaccurate decisions correspond to high baseline charge. In both cases however, the charge achieved in the winning capacitor when the decision is made is constant, and thus the motor implementation of the decision is consistent across decision types.

The electrical circuit described above is a caricature of a real neural decision-making system; it is unlikely that any such system works by translating neural population activation levels directly into motor commands. However if, instead, the output from a neural decision-making population acted as a switch, disinhibiting some motor pathway for example, the same principle of consistency of decision-implementation signal across the full range of decision types would seem useful. To consider such scenarios we can use an electronic analogy, the transistor. Transistors can be used as switches, in just the same way as a neural decision-making population disinhibiting a motor pathway acts as a switch. The current crossing a transistor varies as a saturating function of its controlling input, so when used as switches, the controlling input to the transistor must be either consistently low or consistently high, in order that the current crossing the transistor remains either consistently low or high; intermediate inputs result in intermediate current transfer, so in an electronic circuit emulating the disinhibition of a motor pathway by a neural decision population (figure 3b), consistency of input would also be desirable. The generality of this argument can also be seen without recourse to an electronic analogy. Consider the position of a neural circuit disinhibiting a motor pathway to be that of an observer tasked with deciding whether the signal from a neural decision population means they should or should not disinhibit the pathway; any variability in that signal introduces an unnecessary and undesirable signal detection problem for that observer, and thus introduces an additional potential for mistakes in the sensorimotor pathway.

Having established the general engineering principle, in the next section we briefly review some relevant neurophysiological evidence from the literature.

## Discussion

### Neurophysiological Data on Variable Baseline Activation

A recent review by Bogacz et al. [10] analyses a variety of studies into the neurophsyiological bases of speed-accuracy trade-offs in decision-making. Two of their conclusions are of particular relevance to the hypotheses presented here; first, their survey of three recent fMRI studies [12], [25]–[26], where speed or accuracy was cued in human subjects, concludes that speed-accuracy trade-offs are mediated in the decision-circuits rather than in early sensory or primary motor brain areas; hence mathematical models of the kind shown in figure 1 have the right general structure for formally analysing speed-accuracy trade-offs. Second, these three studies also provide evidence supporting the variable baseline hypothesis; Ivanoff et al. [25] and van Veen et al. [26] both found that cues for speed increased brain activity in frontal and parietal areas including the dorsolateral pre-frontal cortex, while Forstmann et al. [12] found evidence of increased activity in the pre-supplementary motor area. Bogacz et al. [10] concluded that these data directly supporting the variable baseline hypothesis, as well as other data from animals consistent with this hypothesis, combined with a lack of consistent experimental data supporting the variable threshold hypothesis, give a strong overall indication that speed-accuracy trade-offs in decision-making are mediated by changing baseline activation of neural integrator populations. There is also evidence that this variable baseline activation can be implemented by varying levels of input from additional neural populations. For example, Forstmann et al. [27] present evidence that increased connections between pre-supplementary motor area and the striatum perform better in modulating speed-accuracy trade-offs according to instruction, implicating the pre-SMA as providing variable additional input to the striatum to modulate baseline activation.

## Conclusions

We have presented a design principle for how decision-making should be implemented in the brain, and briefly summarised supporting evidence; specifically we propose that decision-making in threshold-based systems should compromise between speed and accuracy of decision-making by manipulation of baseline activation in decision-making neural populations, rather than a manipulation of thresholds, in order to implement stereotypical decisions under varying speed-accuracy tradeoffs. This could be formalised as an optimality argument, that decision-making systems should minimise variability in decision implementation, across decision scenarios; such optimality arguments are commonplace in behavioural disciplines such as behavioural ecology, where their predictions are tested against empirical data, and any disagreement used to refine the theory [28]. In applying this normative approach from evolutionary biology to models of neuroscience, we hope to make a modest contribution to the programme of reconciling functional and mechanistic explanations of behaviour [29]. One potential limitation of our analysis is that equivalence of changes in threshold and changes in baseline activation has only formally been demonstrated for linear models. Real neural systems are typically non-linear, but we argue that even though the aforementioned equivalence does not hold for certain important non-linear models, the principle of maintaining a consistent threshold and varying baseline activation, even if the decision-dynamics are changed as a result and this needs to be compensated for by the neural mechanisms, remains an important one that we should expect to see realised; the neurophysiological evidence supporting this hypothesis, reviewed above, supports this view.

We suggest that our principle is not specific but should be applicable to any response system. Decision-making takes place at many different levels of brain processing, and while more complex decision-related motor sequences undoubtedly can be affected by decision-task difficulty, we believe our principle should also hold at the most fundamental levels of action selection in the brain. Even the conceptually simplest decision-making mechanisms, such as the race model [14] can be expressed as accumulator models. Accumulators are also likely to be involved in more complex decision-making processes; the basal-ganglia have been demonstrated to be involved in action selection, mediating access to motor control by different competing brain regions. A biologically-plausible mathematical model of the basal-ganglia has been proposed that is able to implement statistically optimal decision-making over multiple alternatives [30]. As with the accumulator models outlined above, this model is based on decision-making populations that must reach a threshold in order to precipitate the corresponding action, and this threshold may be adjusted to compromise between the speed and accuracy of decision-making. There is an interesting difference however that, in this model, the output nuclei of the basal ganglia must fall *below* an activation threshold in order for the corresponding action to be taken. However the principle is the same, that in order for consistency of decision-implementation we would expect this threshold to remain constant; therefore we would predict that accurate decisions should be implemented by raised baseline activation levels of decision-making neural populations in the basal ganglia, while fast decisions should be implemented by lower baseline activation. Bogacz *et al.* [10] review four main theories of how speed-accuracy trade-offs can be managed in the cortico-basal ganglia circuit, and note that three involve a change in activation of some part of the circuit, whether striatum [12], cortical integrators [31]–[32], or subthalamic nucleus [33], while none modifies threshold of the output nuclei. We suggest that it could be of interest to interpret not only models but also other data already extant, or yet to be generated, in terms of the proposal we have made here for how consistent decision-implementation should be achieved.

## Methods and Results

### Equivalence of Threshold Change and Baseline Activation Change in the Mutual and Pooled Inhibition Models

Let us denote the activities of the accumulators in the mutual inhibition model (figure 1c) (Usher & McClelland, 2001) by *y*_1_ and *y*_2_. The changes of activity of the accumulators during a small time interval *dt* are given by:
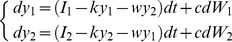
(A1)According to equations A1, each accumulator *i* receives sensory input with mean *I_i_* and noise with magnitude *c* (*dW_i_* denote random numbers from normal distributions with mean 0 and variance *dt*). Furthermore, the activities decay with rate *k*, and each accumulator receives inhibition from the other accumulator weighted by *w*.

To increase the baseline activity of accumulators in a way that it is maintained, an additional input *I*_0_ to both integrators needs to be provided:
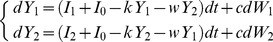
(A2)We use *Y_i_* to denote the activities of integrators in the model with the additional input, to distinguish it from activities of integrators in the model without such input (A.1) which we denoted by *y_i_*. When such an input is provided before stimulus onset (figure 2c), both accumulators converge to the state when the input balances leak and inhibition *Y*_1_ = *Y*_2_ = *I*_0_/(*w*+*k*), which is a fixed point of equations A2 for *I*_1_ = *I*_2_ = *c* = 0.

We now show formally that the model of equations 1 starting at *y*_1_ = *y*_2_ = 0 (illustrated in figure 2a), and model of equations A2 starting at *Y*_1_ = *Y*_2_ = *I*_0_/(*w*+*k*) (illustrated in figure 2d) produce the same shape of trajectories. Note that if the following condition is satisfied (for *i* = 1,2):
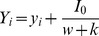
(A3)then *dY*_1_ = *dy*_1_ and *dY*_2_ = *dy*_2_, because substituting equation A3 into the right hand sides of equation A2 gives the right hand sides of equations A1. Therefore the model of equations 1 starting at *y*_1_ = *y*_2_ = 0, and the model of equations A2 starting at *Y*_1_ = *Y*_2_ = *I*_0_/(*w*+*k*) will change the levels of activity of accumulators in exactly the same way in each interval *dt*, and hence the models will produce the trajectories with exactly the same shape but starting from different initial conditions.

The same shape of trajectories of models of equations A1 and A2 shown above also implies that the evolution along the attracting line is unaffected by the additional input. To provide more intuition for this invariance (which may seem surprising) let us note that the position along the attracting line *x* is proportional to the difference between *Y*_1_ and *Y*_2_ (Bogacz et al. 2006):
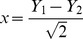
(A4)Taking the derivative of equation A4, and substituting equations A2 we obtain the equation describing the dynamics of the evolution along the attracting line:
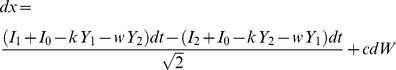
(A5)Note that the increased inputs to both accumulators *I_0_* cancel in equation A5, so the mean drift rate of diffusion along the attracting line and the magnitude of noise in this diffusion are unaffected by *I*_0_, for any parameter values.

Below we show that the same equivalence also holds for the linear version of the pooled inhibition model (figure 1d) (Bogacz et al. 2006). This model consists of two populations of accumulator neurons, which project and receive input from a third population of inhibitory neurons with activity level denoted by *y*_3_. The changes of activity of these neural populations are described by:
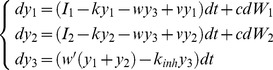
(A6)In equation A6, *k* and *k_inh_* denote the leak of accumulators and inhibitory population respectively, *w* and *w′* are respectively the weights of connection from inhibitory population to integrators and vice versa, and *v* is the weight of the self-excitatory connection within each neuronal population corresponding to an accumulator. The common input to both accumulators can be introduced as in the mutual inhibition model:
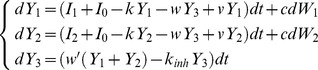
(A7)Analogously as for the mutual inhibition model, if the common input is provided before the start of the decision (i.e. when *I*_1_ = *I*_2_ = *c* = 0), the activities of populations will converge to a fixed point of equation A7:
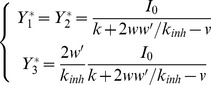
(A8)Analogously as for the mutual inhibition model, since *Y_i_* = *y_i_*+Y_i_*, then *dY_i_* = *dy_i_*, which implies that adding common input to accumulators before and during the choice process will simply shift the trajectories towards the thresholds, thus it will have exactly the same effect on accuracy and reaction time as lowering the thresholds.
